# Supplementary material for: Impact of mass administration of azithromycin as a preventive treatment on the prevalence and resistance of nasopharyngeal carriage of Staphylococcus aureus
Source: PLoS One. 2021 Oct 13;16(10):e0257190. doi: 10.1371/journal.pone.0257190 (PMC8513893; doi:10.1371/journal.pone.0257190)
Supplement: S1 File — (DOC) [file pone.0257190.s001.doc]

**
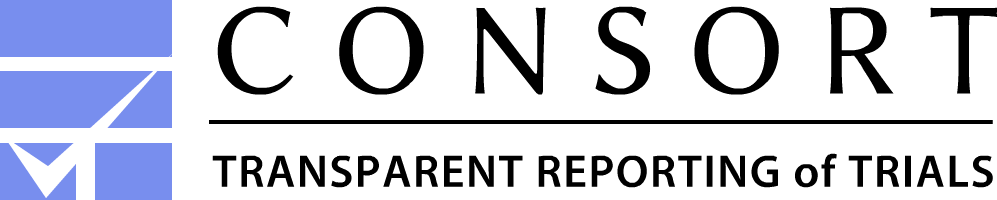
**

**CONSORT 2010 Flow Diagram**

**Allocation**

**Analysis**

**Follow-Up**

**Enrollment**

Assessed for eligibility in 2016 (n= 880)

Excluded (n= 0)

♦  Not meeting inclusion criteria (n=0)

♦  Declined to participate (n= 55)

♦  Refusal to take a sample (n=47 )

Analysed (n=385)
♦ Excluded from analysis (n= 0)

Lost to follow-up (give reasons) (n= 0)

Discontinued intervention (give reasons) (n= NA)

Nasopharyngeal swabbing before administration of Azithromycin or placebo (n=385)

♦ Children AZ (n=193)

♦ Children P (n= 192)

Lost to follow-up (give reasons) (n=0)

Discontinued intervention (give reasons) (n=NA)

Nasopharyngeal swabbing after administration of Azithromycin or placebo (n=393)

♦ Children AZ (n=192)

♦ Children P (n= 201)

Analysed (n=393)
♦ Excluded from analysis (n=0)

Randomized for Nasopharyngeal swabbing in 2016 (n=778)
