# Supplementary material for: Impact of mass administration of azithromycin as a preventive treatment on the prevalence and resistance of nasopharyngeal carriage of Staphylococcus aureus
Source: PLoS One. 2021 Oct 13;16(10):e0257190. doi: 10.1371/journal.pone.0257190 (PMC8513893; doi:10.1371/journal.pone.0257190)
Supplement: S2 File — (PDF) [file pone.0257190.s002.pdf]

STUDY PROTOCOL

A trial of seasonal malaria chemoprevention plus azithromycin in African children

Protocol Number: 4

Brief Title: SMC+AZ Trial

Protocol Version & date: Version 4 – (9 July 2014)

Sponsor: London School of Hygiene & Tropical Medicine,
Keppel Street, WC1E 7HT,
London

Lead Principal Investigators: Daniel Chandramohan & Brian Greenwood

Site Study Directors: Jean Bosco Ouedraogo & Ogobara Doumbo

Contact details of the principal Investigators

Daniel Chandramohan	London School of Hygiene & Tropical Medicine, Keppel St., London WC1E 7HT, UK	Tel: + 44 207 9272322<br>E-mail: daniel.chandramohan@lshtm.ac.uk
Brian Greenwood	The London School of Hygiene & Tropical Medicine, Keppel St., London WC1E 7HT, UK	Tel: + 44 207 2994707<br>E-mail: brian.greenwood@lshtm.ac.uk

Contact details of site study directors

Jean Bosco Ouedraogo	Centre Muraz<br>IRSS/Centre Muraz,<br>Institut de Recherche en	Tel (226) 20981880<br>Tél/Fax (226) 20974868<br>E-mail: jbouedraogo.irssbobo@fasonet.bf
	Sciences de la Santé, Direction Régionale de l'Ouest (IRSS-DRO)<br>399 Avenue de la Liberté<br>01 BP 545 Bobo-Dioulasso 01, Burkina Faso	or jbouedrago@gmaim.com
Ogobara Doumbo	Malaria Research & Training Center; University of Sciences Techniques and Technologies of Bamako, P.O. Box 1805<br>Bamako, Mali	Tel: +223 2022 8109<br>E-mail: okd@icermali.org

Contact details of site principal investigators in Burkina Faso

Tinto Halidou	Unité de Recherche Clinique de Nanoro (URCN)<br>Institut de Recherche en Sciences de la Santé, Direction Régionale de l'Ouest (IRSS-DRO)<br>399 Avenue de la Liberté<br>01 BP 545 Bobo-Dioulasso 01, Burkina Faso	Tel. +226 70346354 or +226 76541180<br>E mail. tintohalidou@yahoo.fr or tintoh@fasonet.bf
Issaka Zongo	Institut de Recherche en Sciences de la Santé, Direction Régionale de l'Ouest (IRSS-DRO)<br>399 Avenue de la Liberté<br>01 BP 545 Bobo-Dioulasso 01, Burkina Faso	Tel : (00226) 20 981 880<br>Email: issaka.zongo@lshtm.ac.uk or zongo_issaka@yahoo.fr

Contact details of site principal investigators in Mali

Alassane Dicko	Malaria Research & Training Center; Department of Epidemiology of Parasitic Diseases; Faculty of Medicine, Pharmacy and Dentistry; University of Sciences Techniques and Technologies of Bamako, P.O. Box 1805 Bamako, Mali	Tel: +223 2022 8109<br>E-mail: adicko@icermali.org
Issaka Sagara	Malaria Research & Training Center; Department of Epidemiology of Parasitic Diseases; Faculty of Medicine, Pharmacy and Dentistry; University of Sciences Techniques and Technologies of Bamako, P.O. Box 1805 Bamako, Mali	Tel: +223 2022 8109<br>E-mail: isagara@icermali.org

Contact details of other investigators and collaborators

Paul Milligan	London School of Hygiene & Tropical Medicine, Keppel St., London WC1E 7HT, UK	Tel: + 44 207 927 2126<br>E-mail: paul.milligan@lshtm.ac.uk
Matthew Cairns	London School of Hygiene & Tropical Medicine, Keppel St., London WC1E 7HT, UK	Tel: + 44 207 9588104<br>E-mail: matthew.cairns@lshtm.ac.uk
Simon Cousens	London School of Hygiene & Tropical Medicine, Keppel St., London WC1E 7HT, UK	Tel: + 44 207 927 2422<br>E-mail: simon.cousens@lshtm.ac.uk
Irene Kuepfer	London School of Hygiene & Tropical Medicine, Keppel St., London WC1E 7HT, UK	Tel + 44 207 9272322<br>Email: irene.kuepfer@lshtm.ac.uk
Lesong Conteh	Imperial College, School of Public Health,Centre for Health Policy<br>Sherfield Building<br>South Kensington Campus<br>London	Tel: +44 (0)20 7594 5029<br>E-mail: l.conteh@imperial.ac.uk
Yves Daniel Compaoré	Institut de Recherche en Sciences de la Santé, Direction Régionale de l'Ouest (IRSS-DRO)<br>399 Avenue de la Liberté<br>01 BP 545 Bobo-Dioulasso 01, Burkina Faso	tél : (00226) 20 981 880<br>Email : yvesdaniel.co@gmail.com
Abdoulaye Djimde	Malaria Research & Training Center; Department of Epidemiology of Parasitic Diseases, University of Sciences Techniques and Technologies of Bamako, P.O. Box 1805 Bamako, Mali	Tel: +223 20220 8109<br>E-mail: adjimde@icermali.org
Samba O Sow	Center for Vaccine Development – Mali; CNAM; Bamako, Mali	Tel: +223 6674 8947<br>E-mail: ssow@medicine.umaryland.edu

The signatures below confirm agreement by the individuals authorised by the sponsor and principal participating institution at the clinical site that the study will be conducted in compliance with protocol version 4 dated 9th July 2014.

………………………………………	……………………………..	………
Study Director in Mali	NAME (PRINTED)	DATE
………………………………………	……………………………..	………
Study Director in Burkina Faso	NAME (PRINTED)	DATE

List of Abbreviations

ACT… ....Artemisinin combination therapy

AFRO......... African regional office of WHO

AQ .... Amodiaquine

AZ...............Azithromycin

CIPLA ……The Chemical, Industrial, & Pharmaceutical Laboratories, India

GCP........... Good clinical practice

IDMC ....Independent Data Monitoring Committee

ITN............. Insecticide-treated bednet

LDH .... Lactic dehydrogenase

LSHTM ..... London School of Hygiene & Tropical Medicine

MDG ....Millennium Development Goals

MMV...........Medicines for Malaria Venture

MPAC ....Malaria Policy Advisory Committee (WEHO)

NMCP ....National Malaria Control Programme

SMC.......... Seasonal Malaria Chemoprevention

RDT. ....Rapid Diagnostic Test

SP............. Sulphadoxine/pyrimethamine

WAHO ......West Africa Health Organization

WHO......... World Health Organization

Protocol Summary

**Title**	A trial of seasonal malaria chemoprevention plus azithromycin in African children.
**Study objective**	The primary objective of this study is to determine whether addition of azithromycin (AZ) to Seasonal Malaria Chemoprevention (SMC) using sulphadoxine/pyrimethamine (SP)+amodiaquine (AQ) will provide an additional reduction in deaths and severe illness in young African children. The secondary objectives include an assessment of the safety and cost-effectiveness of the addition of AZ to SMC with SP+AQ.
**Study design**	The study will be a double blind, randomised, placebo controlled trial. The unit of randomisation will be the household. Children aged 3 - 59 months will be randomised to receive four cycles of either SP+AQ+AZ or SP+AQ+ placebo at monthly intervals during the peak malaria transmission season.
**Study site**	The trial will be conducted in Hounde district in Burkina Faso and in Bougouni district, Mali.
**Study population**	Children of either sex aged 3-59 months of age at the start of each period of drug administration will be eligible for inclusion in the trial provided that parental consent is obtained. Children with a severe, chronic illness or known allergy to one of the study drugs will be excluded.
**Control arm**	Children in the control group will be given SP+AQ plus a matching placebo for AZ by mouth monthly for four months during the high malaria transmission season (August to November).
**Intervention arm**	Children in the intervention group will receive SP+AQ plus AZ by mouth monthly for four months during the high malaria transmission season.
**Primary endpoint**	Incidence of the combination of death or hospital admission for at least 24 hours, not due to trauma or elective surgery during the intervention period.
**Secondary endpoints**	(a) incidence of the combination of death or hospital admission for at least 24 hours not due to trauma or elective surgery during the whole study period (intervention and post-intervention periods),<br>(b) attendance at a study health centre with a febrile illness that is not due to malaria (including acute respiratory infections and diarrhoea),<br>(c) attendance at a study health centre with RDT or microscopically proven malaria,<br>(d) the prevalence of moderate anaemia (Hb <8 g/dL) at the end of each malaria transmission season,<br>(e) nutritional status at the end of each malaria transmission season,<br>(f) the prevalence of nasopharyngeal carriage with pneumococci before and at the end of each malaria transmissions season,<br>(g) the prevalence of carriage with macrolide resistant pneumococci before and at the end of each malaria transmission season,<br>(h) the prevalence of resistance markers to SP in children with *Plasmodium falciparum* malaria at the end of the study,

	(i) the effect of the addition of AZ to adherence with SMC.
**Sample size**	A sample size of 19,200 children (9600 in each country) followed for three malaria transmission seasons and two dry seasons will give a study which has 90% power to detect a reduction of 25% in hospital admissions and all cause deaths, and 80% power to detect a reduction of 22%.
**Study duration**	Jan 2014 – June 2017

Table of Content

1. Background and rationale……………………………………………………….. 7
2. Study objectives…………………………………………………………… 9
3. Study sites …………………………………………………………………. 9
4. Methods…………………………………………………………………. 10
   4.1. Study design………………………………………………………… 10
   4.2. Community consent.. 10
   4.3. Census and randomisation ………………….. ………………………. 10
   4.4. Inclusion and exclusion criteria ………………………………………… 11
   4.5. Sample size ………. …………………………………………………….. 11
   4.6. The intervention ………………………… ……………………………. 12
   4.7. Follow up and measurement of outcomes …………………………… 13
   4.8. Laboratory procedures …………………………………………………… 14
5. Socio-economic studies ………………………………………………… 15
   5.1. Objectives ...., ...15
   5.2. Economic evaluation ....,........... 15
   5.3. Equity implications...16
   5.4. Modelling affordability and sustainability of SMC at scale..........................16
6. Monitoring and quality assurance …………………………………………… 16
7. Data management and analysis plan ………………………………………. 17
8. Safety and ethical considerations ……………………………………………. 17
9. Trial sponsors and management ……………………………………………. 18
10. Roles of the investigators and collaborators 19
11. Stakeholder engagement and dissemination plans..19
12. Activity Schedule ………………………………………..………………… 21
13. References …………………………………………………………………….. 22
14. Appendices
   Appendix 1: Calculation of design effect. ……………………………………. 23
   Appendix 2: Sample size estimates..24
   Appendix 3: Definitions of adverse events and serious adverse events
   and reporting schedule ………………………………………… 26

Background and rationale

Child mortality continues to be unacceptably high in many countries in sub-Saharan Africa and these countries are unlikely to meet Millennium Development Goal (MDG) 4, reducing child mortality by two thirds from its 1990 level, by 2015 unless novel approaches to the control of early childhood deaths are developed. In recent years, several countries in sub-Saharan Africa have established successful malaria control programmes and these have been accompanied by substantial reductions in overall child mortality. However, in many parts of Africa, including Burkina Faso and Mali, control of malaria has been more limited despite widespread deployment of effective control measures such as insecticide treated bed nets (ITNs), Rapid Diagnostic Tests (RDTs) and artemisinin combination therapy (ACT). In countries such as these, new approaches to malaria control are needed. One of these is Seasonal Malaria Chemoprevention (SMC).

SMC involves administration of an antimalarial drug or drug combination in a full therapeutic course to children on three of four occasions during the period of highest risk of malaria infection. Studies undertaken in several countries in West Africa, including Burkina Faso and Mali, have shown that SMC with sulphadoxine/pyrimethamine (SP) and amodiaquine (AQ) is highly effective in areas where the transmission of malaria is markedly seasonal, reducing the incidence of severe and uncomplicated malaria by up to 80% [1-3]. SMC has reduced overall hospital admissions by 40% and, in some studies, reduced the prevalence of anaemia and stunting [1]. No individual study has been large enough to measure an impact on mortality but the combined results of six controlled trials suggest a reduction in mortality in children aged 3 - 59 months of about 35%, although this estimate has wide confidence intervals. SMC with a combination of SP and AQ is safe, with no serious drug related adverse event being reported after administration of over 800,000 courses in Senegal [4]. SMC can be delivered effectively by community health workers [5, 6], its administration can readily be combined with community treatment of clinical episodes of malaria [7] and it is highly cost effective [8]. Recent studies have defined the areas where SMC would be an appropriate intervention based on the seasonality and incidence of malaria [9]. These include most of the Sahel and sub-Sahel, population approximately 150 million, and possibly other areas in southern and eastern Africa.

A Technical Expert Group of the WHO reviewed all the available evidence on the efficacy and safety of SMC in May 2011 and recommended SMC with SP+AQ in areas of the Sahel and sub-Sahel with marked seasonal transmission of malaria. This recommendation was endorsed at the first meeting of the WHO Malaria Policy Advisory Committee (MPAC) in February 2012. Most countries in the Sahel and sub-Sahel region have incorporated SMC, along with other malaria control interventions, in their strategic malaria control plan and implementation

documents have been developed and disseminated by WHO [10]. Pilot implementation studies were undertaken in Mali and in Chad in 2012 by Médecins sans Frontières and also in Niger, Nigeria and Togo in 2013.More widespread implementation of SMC is likely in 2014.

In most countries in Africa, acute bacterial infections account for as many deaths and episodes of severe illness as malaria [11] and this proportion will increase as malaria is increasingly brought under control. Preventing deaths from acute bacterial infections is key to achieving MDG4. Increasing deployment of pneumococcal conjugate vaccines and improved case management will help in achieving this goal but other, novel, approaches are needed. In high risk areas, one of these might be chemoprevention used in a manner analogous to that now being promoted for the prevention of malaria. Results of a recent study from Ethiopia suggest that this might be feasible [12,13] but this needs to be confirmed.

Mass treatment with azithromycin (AZ) is a highly effective approach to the control of trachoma and, in some areas, has led to local elimination of this infection [14]. A surprising finding that emerged from an AZ mass trachoma treatment programme undertaken in Ethiopia was that administration of this drug on a single occasion was associated with a substantial reduction in overall child mortality. This reduction was sustained for 26 months after treatment when the Rate Ratio (RR) for death in children who had received AZ was still substantially lower at this time than in those who had not received AZ (RR 0.35; 95% C1: 0.17, 0.74) [13]. It is not clear how this effect was achieved. AZ is an antimalarial, although only a weak one when used alone [15], so it is possible that the effect was achieved partly by preventing malaria. However, AZ is also a highly effective broad spectrum antibiotic so that its impact on mortality could have been achieved through its action on pneumococci and other bacteria, for example by clearing pneumococcal carriage. If SMC is implemented in countries of the Sahel and sub- Sahel as a malaria control measure, it would be possible to add AZ to the antimalarial drug regimen given during the rainy season when the risk of malaria is highest but which is also the time of the year when the incidence of severe bacterial infections is at its peak. Once a delivery system has been established to deliver SMC, the only significant additional cost would be that of the AZ, which is now available in generic form at a reasonable cost. Therefore, we propose to investigate whether adding AZ to an SMC regimen would provide additional benefitby providing protection against common bacterial infections as well as against malaria.

1. Study objectives and outcomes

The primary objective of this study is to determine whether addition of AZ to SMC using SP+AQ will provide an additional reduction in deaths and severe illness in young African

children. The secondary objectives of the trial include assessment of the safety and cost-effectiveness of the addition of AZ to SMC with SP+AQ.

The primary outcome measure is the combined incidence of hospital admissions for at least 24 hours, not due to trauma or elective surgery, and death from any cause other than external causes during the intervention period.

Secondary trial end points will include –

(a) Incidence of hospital admissions and deaths, as described above, over the entire study period (intervention and post-intervention periods),
(b) attendance at a study health centre with a febrile illness that is not due to malaria including episodes of acute respiratory infection and diarrhoea,
(c) attendance at a study health centre with microscopically proven malaria,
(d) the prevalence of malaria parasitaemia at the end of each malaria transmission season,
(e) the prevalence of moderate anaemia (Hb <8 g/dL) at the end of each malaria transmission season,
(f) nutritional status at the end of each malaria transmission season,
(g) the prevalence of nasopharyngeal carriage with pneumococci before and at the end of each malaria transmissions season,
(h) the prevalence of carriage with macrolide resistant pneumococci before and at the end of each malaria transmission season,
(i) the prevalence of resistance markers to SP in children with *Plasmodium falciparum* malaria at the end of the study,
(j) the effect of the addition of AZ to adherence with SMC.

2. Study sites

The trial will be conducted in Hounde health district, Burkina Faso and in Bougouni Koulikoro district, Mali. The Hounde district is served by one district hospital and is situated 300 km from Ouagadougou and 100 Km from Bobo-Dioulasso where the CHUSS, the 2nd National Reference Hospital (University Hospital) is located. The study site in Mali is the district of Bougouni in the region of Sikasso, Mali, 150 km south of Bamako.

3. Methods

4.1. Study design

The study will be a double blind, randomised, placebo controlled trial. The unit of randomisation will be the household. Households with 3 - 59 month old children will be randomised to receive four cycles of either SP+AQ+AZ or SP+AQ+ matching placebo at monthly intervals during the malaria transmission season.

4.2 Community permission

The objectives of the study and the way in which it will be conducted will be discussed with Ministry of Health and district health officials prior to its commencement. Community approvalwill be sought through meetings with leaders of the study communities and through open meetings held in the study communities. Community leaders will be consulted prior to the start of the intervention on the best ways of achieving high compliance with drug delivery and follow-up.

4.3. Census and randomisation

Once community permission for the trial has been obtained, a household census will be conducted and all households within the study areas with children <5 year of age will be identified. At the census, a preliminary screening of potentially eligible children will be undertaken. Eligible children (see below) and their caretakers will be visited again and written informed consent will be obtained from their caretakers for their inclusion in the trial before the administration of first round of study drugs. Children entered into the trial will be assigned a unique ID number and their demographic data (date of birth and/or age, and gender) will be collected. The census will be updated at the start of each malaria transmission season and potentially eligible new trial participants in households already included in the trial and in new households will be identified and screened for their eligibility to join the trial.

The unit of randomisation will be the household and all eligible children from a household will be allocated to the same study group to avoid confusion. Sharing a kitchen will be used to define a household. Households with at least one potentially eligible child will be randomised to the two study groups using permuted blocks. Any new households enrolled at the start of year 2 or the start of year 3 will be randomised similarly.

4.4 Inclusion and exclusion criteria

Children of either sex aged 3-59 months of age at the start of each period of drug administration will be eligible for inclusion in the trial provided that parental consent is obtained. Children under the age of three months on the date of the start of drug administrationwill be excluded that year as SMC is not recommended for children in this age group whose risk of acquiring malaria is low. Children who reach the age of 59 months or more at the end of the first or second years of treatment will not receive SMC in subsequent years.

Exclusion criteria will be as few as possible to make the results of the trial broadly applicable and will be only (a) a severe, chronic illness, (b) a known allergy to one of the study drugs. (c) HIV+ children on cotrimoxazole prophylaxis, Children with an acute illness at the time that drug administration is due will be excluded temporarily and will not be given SMC but will be given an appropriate treatment for their illness. However, they will be eligible for SMC during subsequent cycles.

4.5 Sample size

The sample size for the primary endpoint was derived using methods for cluster-randomized trials with incidence rates as the outcome, assuming follow-up over a three-year period in both Burkina Faso and in Mali.

The mechanism by which AZ reduces morbidity and mortality may be by clearing pneumococci from the nasopharynx. Thus, randomisation by household rather than by individual will help to minimize dilution of the impact of AZ through re-infection of study children receiving AZ from their untreated siblings. The design effect resulting from household randomisation is expected to be at most 1.2 (details shown in appendix 1) implying that a sample size increase of 20% would be sufficient to account for the intra-household correlation.

On the basis of data from large SMC trials conducted in Burkina Faso [3] and Mali [2] and data from a large trial of a nine-valent pneumococcal conjugate vaccine in The Gambia [16], we have assumed that the incidence of hospital admissions and deaths (excluding trauma) during the malaria transmission season will be at least 15 per 1000 children in the SP+AQ only group. Based on our experience with previous trials in Burkina Faso and Mali, we assumed that 10% of households will be lost to follow up each year. Drop-out is assumed to occur on average in the middle of the transmission season so that the expected follow up time contributed by a household is 0.95 of a transmission season in year 1, 0.855 in year 2 and 0.7695 in year 3, a total of 2.57. Therefore, 2.57 transmission seasons has been used as the average duration of follow-up over a three year study period.

With these assumptions, 19,200 children (9600 in each country) followed for three malaria transmission seasons and two dry seasons would give a study which has 90% power to detect a reduction of 25% in hospital admissions and all cause deaths, and 80% power to detect a reduction of 22%. Should the incidence of deaths and hospital admissions be lower than we have anticipated at 10 per 1000 children followed during the malaria transmission season, the study will have 90% power to detect a protective effect of 30%, and 80% power to detect a protective effect of 26%. A range of sample size estimates based on various assumptions are shown in appendix 2.

Children who have reached the age of five years at the time of the first cycle of treatment in the second and third year will not receive further treatment but will be followed up until the end of the study. Newborns from the study area will be recruited into the study when they reach the age of 3 months. Thus, the number of children lost at the age of five years will be balanced approximately by the number of newly recruited infants.

At the end of the first year, the sample size will be re-calculated, without unblinding, using the estimates of event rates in year 1.

4.6 The intervention

Children in the control group will be given SP+AQ plus a matching placebo for AZ and children in the intervention group will receive SP+AQ plus AZ, monthly for four months during the high malaria transmission season (August to November). All treatments will be given by mouth. Blister packs of SP+AQ+AZ and SP+AQ+AZ placebo will be obtained from CIPLA, India or through MMV. Blister packs of SP+AQ+ AZ will be made for two different age groups. The dose regimen for infants (3-11 months) is S/P 250mg/12.5 mg and AQ 75mg on day 1 and AQ 75mg on days 2 and 3; for children 1-4 years it is S/P 500mg/25 mg and AQ 150mg on day 1 and AQ 150mg on days 2 and 3. The dose regimen of AZ for infants (3-11 months) will be 100mg on days 1, 2, and 3; for children (1-4 years) it will be 200mg on days 1,2, and 3. The placebo of azythromycin will have the same composition as azythromycin tablets but without the active ingredient. The inactive ingredients may include calcium phosphate, magnesium stearate, titanium dioxide and triacetin. The composition of the placebo will be forwarded to the IRB as soon as it is confirmed by the manufacturer.

Treatment with each dose will be given by trained, paid volunteers at a central point in each study community under observation. Home visits will be made to children who miss treatment and their parents/guardians will be asked if they would like their child to receive the drugs. If they agree, treatment will be given at home. Drugs will be pre-packed in re-sealable envelopes bearing the child's unique number and containing tablets for four cycles of treatment required for one full malaria transmission season (and repeat doses in case these are required) appropriate for the child's age.

4.7 Follow-up and measurement of outcomes

Deaths occurring in the study health facilities or in the study communities will be recorded throughout the study period by project staff. The vital status of study children will be monitored at the annual pre-intervention census, household visits for active morbidity surveillance, and at the annual end of transmission season cross sectional surveys. Verbal autopsies will be carried out to determine the causes of death.

Members of the study teams, who will be blind to treatment allocation, will be based at health centres and in district hospitals where study children are likely to present if they are ill. Mothers will be encouraged to bring their child with the ID card to the health centre if their child is unwell and told that the costs of treatment will be provided by the project. A record will be kept of the attendance of any study child at a peripheral health facility by dispensary staff and the nature of their illness will be recorded on a standardised case reporting form. Outpatient episodes of acute respiratory infections and diarrhoeal illness will be carefully defined. Notes will be made of vaccines that a child has received, in particular whether a child has received a pneumococcal vaccine. Children with an illness suggestive of malaria will be tested for malaria with an RDT, provided by the project, to guide treatment, and subsequently by microscopy at a central laboratory to increase diagnostic accuracy. Referral to hospital or a community health centre will be facilitated by the provision of transport costs for those with a severe illness. Illnesses in study children admitted to hospital will be investigated by medical staff following standardised operating procedures and using a standardised case report form. Each week during the malaria transmission season, 100 randomly selected children in each country will be visited at home and a blood film collected to provide information on the prevalence of malaria infection.

Home visits will be made seven days after drug administration in a randomly selected group of 400 children in each country after each round of drug administration in the first year of the study to record adverse events. One hundred of these children will be visited after each round of treatment in case there are any side effects resulting from drug accumulation. Surveillance of all children will be maintained throughout the study period for any drug-related Serious Adverse Events (SAEs). Details of definitions of adverse events and SAEs and of reporting procedures are described in appendix 3.

At the end of each malaria transmission season, a cross-sectional survey will be undertaken in a sample of 2,000 children in each country, randomly selected at the time of enrolment, during which they will be examined, their nutritional status recorded and a finger prick blood sample obtained for measurement of haemoglobin concentration, preparation of a blood film for microscopy and collection of a blood spot for molecular analyses. In addition 500 randomly selected school children aged 5-12 years resident in the study area who are well and have not

received SMC will be tested for malaria by RDT, microscopy and PCR at the end of each malaria transmissions season to determine the overall level of malaria transmission and the trend in the distribution of molecular markers of resistance to SP in the study area during the period of the trial.

Nasopharyngeal swabs will be collected from 400 children (200 in each group) in each country before the first administration of SMC and during the cross-sectional surveys at the end of each intervention period for isolation of pneumococci and antibiotic sensitivity testing of pneumococcal isolates. A final survey will be undertaken 6 months after the final drug administration if a significant increase in macrolide resistance is found at the post-implementation survey in year three of the study.

For each of the surveys described above, a random sample of households in each arm of the trial will be identified at randomisation, and one child per household selected at random to be sampled.

4.8 Laboratory Procedures

4.8.1 Detection of malaria

A histidine rich protein (HRP2) based RDT will be used for the initial diagnosis of malaria and to guide treatment. Blood films collected at the same time will be read subsequently by two microscopists. All slides will be read twice by two separate readers. Slides which are judged to be discordant for either positivity or parasite density will be read by a third reader. For slides with high or medium density parasitaemia (> 400/µl) readings will be considered discordant if the higher count divided by the lower count is > 2. In the case of slides with low density parasitaemia (< 400/µL), readings will be considered discordant if the highest reading densityis more than one $\log_{10}$ higher than the lowest reading. In cases when one reader gives a count > 400/µL and the other < 400/µL, the second criterion will apply. For cases of discrepancy in definition of positivity/negativity, the majority decision will be adopted. If the majority decision is positive, the final result will be the geometrical mean of the two positive readings. In the case of discrepancies in parasite density, the final result will be the geometric mean of the two geometrically closest readings.

4.8.2 Detection of markers of resistance to SP

Nested PCR reactions will be used to detect the presence of mutations in the *dhfr* and *dhps* genes associated with resistance to pyrimethamine and sulphadoxine respectively as described previously [17].

4.8.3 Measurement of haemoglobin concentration

Haemoglobin concentration will be measured colorimetrically using a Hemocue colorimeter (Hemocue AB, Angelholm, Sweden).

4.8.4 Isolation of pneumococci and testing for macrolide resistance

Nasopharyngeal swabs (NPS) will be collected, using a calcium alginate swab, from the posterior wall of the nasopharynx and immediately inoculated into vials containing skim milk-tryptone-glucose-glycerol (STGG) transport medium. Vials will be placed in a cold box before transfer to the laboratory within eight hours of collection, in accordance with the WHO protocol for evaluation of pneumococcal carriage, and vials were stored at -80° C prior to analysis. Ten µl of thawed, inoculated STGG medium will be plated onto a gentamicin blood agar plate and incubated for 18-24 hours at 35° C in 5% CO_2. Pneumococcal identification will based on colony morphology and conventional methods of characterization (optochin susceptibility and bile solubility assays). Antimicrobial sensitivity will be measured using antibiotic impregnated discs. Penicillin and macrolide resistance will be confirmed using E-test strips.

5 Socio-economic studies

5.1. Objectives

The objectives of the socio-economic studies are -

1. To estimate and compare the cost effectiveness of the two interventions (SMC vs SMC+AZ) by combining epidemiological outcomes with costs to both the health system (including activities associated with SMC at the community level and within health facilities) and to households.

2. To investigate the equity implications of SMC vs SMC+AZ.

3. To explore the affordability and sustainability of SMC+AZ at scale.

5.2. Economic Evaluation

Cost effectiveness ratios will be presented from three perspectives (i) intervention costs alone, (ii) potential costs/savings to health providers (MoH), (iii) potential societal costs/savings, which includes both health providers and households. The economic evaluation will explore at which point SMC+AZ is no longer cost effective given changing epidemiological and economic parameters, using a threshold analysis

Costs to the health care providers and households associated with SMC with or without AZ will be collected as part of routine trial data collection where possible, with supplementary cost and resource use data collected when necessary. In addition, the economic costs to providers of treating malaria in children will be based on detailed cost data from a representative subsample of health facilities. A standardised costing template will be used in all the sites to

record resource use associated with personnel, materials and supplies, equipment, transport, utilities and buildings. A standard ingredients approach will be used which involves costing the quantity used and the value of each unit of input needed to provide an inpatient or outpatient visit.

Effects will be based on trial outcomes. Specifically, the cost per all cause deaths or hospital admissions (excluding trauma) averted will be calculated.

5.3. Equity implications

Data on the socio-economic characteristics of study participants will be collected as part of the wider trial. These data will be analysed in conjunction with process indicators (such as children receiving full, moderate, low or no doses) and health outcomes (such as deaths averted) across different socio-economic sub-groups in each trial arm.

5.4. Modelling affordability and sustainability of SMC at scale

Whether an SMC + AZ programme would be affordable to both the heath care provider – namely the Ministry of Health - and to the caregivers over a wide range of budgets will be explored. A simple model forecasting the costs of introducing and sustaining AZ and SMC will be developed and this will run using various ‘delivery’ (supply) and ‘uptake’ (demand) scenarios. This model will help to inform decision makers about the affordability of the combining the delivery of AZ and SMC in their settings.

6. Monitoring and quality assurance

An independent GCP monitor appointed by the sponsor (LSHTM) will ensure the quality of the data collected and that GCP standards are met. The monitor will conduct a trial initiation visit, a close out visit and at least one additional visit each year. The monitor will ensure that the trial is conducted according to the study protocol, that appropriate ethical procedures are in place and s/he will examine a random selection of clinical and laboratory records during each visit.

7. Data management and analysis

Data will be managed using the DataFax system. The system is based on faxing the CRFs from the research sites and the data are automatically captured and validated. The MRTC data management team will be responsible for the training of the IRSS data management team with the support of the NIAID/NIH central data management team. A formal analysis plan will be prepared and approved by the Independent Data Monitoring Committee (IDMC) appointed for the trial before the study code is broken. Both intention to treat and per protocol analyses will be undertaken. Children who received any dose of SMC will be included in the intention to treat analysis. Children who received at least the first dose of all three treatment courses will

be included in the per protocol analysis for each year of the study. Results obtained in Burkina Faso and Mali will be analysed separately but the study is only powered to meet its primary and major secondary end-points if results from both countries are combined. Additional sub-analyses will include analysis by age, bed net use and socio-economic status.

Depending upon the number of events recorded during the first year of the trial, the IDMC will consider whether an interim analysis at the end of the second year would be appropriate.

Once the analyses set out in the analysis plan has been completed, data from the trial will be made available to legitimate investigators for further analysis provided that approval is given for this by (a) the principal investigator for each centre and (b) the ethics committees that were responsible for the initial approval of the trial.

8. Safety and ethical aspects of the project

Risks to participants in the trial are small. The trial will use only licensed products and does not involve any new methods. Over 800,000 courses of SP and AQ have been given for SMC in Senegal without any report of a drug associated severe adverse event (SAE), despite enhanced surveillance. Similarly, millions of treatments with AZ have been used in trachoma elimination programmes without reports of any serious toxicity [18]. Pfizer has recently developed a fixed combination of AZ and chloroquine and shown this to be a safe and reasonably effective antimalarial and therefore no adverse events or interactions are anticipated from co-administration of SP+AQ+AZ. Both AQ and AZ can cause nausea and vomiting in some recipients but this has not proved to be a problem for compliance in either SMC or AZ mass treatment programmes. The incidence of adverse events will be monitored in a randomly selected group of 400 children on day 7 post administration of SMC or SMC+AZ after each round in the first year of the trial. All serious adverse events will be monitored continuously throughout the study period through the recording of all hospital admissions and deaths. A monitoring of the pharmacovigilance integrated into the current health system will be established in accordance with the national and the WHO guidelines for SMC implementation.

The details of the definitions and reporting procedures of adverse events and SAEs are described in appendix 3.

The main potential hazard from the intervention is the possible impact of large scale drug administration on the drug sensitivity of malaria parasites and potentially pathogenic bacteriasuch as the pneumococcus.

The possibility that SMC with SP+AQ might induce resistance tothese drugs in *P. falciparum* has been investigated in a number of trials of SMC. Selection ofparasites carrying mutations which confer resistance to pyrimethamine or sulphadoxine has been demonstrated in some but not all studies [1]. However, because the prevalence of parasitaemia in children who received SMC was substantially less than in the control group, the total number of parasites carrying resistance markers was less in children who had received SMC than in control children. Extensive use of SP for intermittent preventive treatment in pregnant women has not accelerated resistance to SP in West Africa. The potential risk of inducing resistance to SP or AQ by SMC was reviewed carefully by the WHOTechnical Expert Group and considered to be an acceptable risk in light of the major benefitsconveyed by the intervention. Malaria parasites isolated at the end of the transmissions season will be tested for resistance markers to SP.

Widespread deployment of AZ carries the risk of inducing macrolide resistance in *Streptococcus pneumoniae* and perhaps in other bacteria. This has been noted in some trachoma elimination programmes [19] but not in others [20] and the effect has usually been transitory. In light of this potential risk, pneumococci will be tested for macrolide and penicillin resistance at the end of each year of the intervention. If macrolide resistance is found at the end of the trial, a further carriage study will be undertaken 4-6 months later to determine if this was only a transitory phenomenon as has been noted following mass AZ treatment programmes for trachoma.

Conduct of the trial will not impose any additional costs on the local health services. The project will contribute to the costs of routine clinical care of study subjects during the trial andto strengthening the district hospitals in the study areas. Individual, written, informed consentwill be obtained from the family of each child entered into the trial. Ethical approval will be obtained from the Ethics Committee of LSHTM, the Health Research Ethics Committee of Burkina Faso, the Institutional Ethics Committee of Centre Muraz in Burkina Faso and the Ethics Committee of the Faculty of Medicine, Pharmacy and Dentistry, University of Bamako.

9. Trial sponsors and management

The trial will be sponsored by the London School of Hygiene & Tropical Medicine which has a dedicated clinical trials department.

An independent trial steering committee, which will provide scientific oversight, will be established and their approval of the protocol will be obtained. The steering committee will hold teleconferencing or face-face meeting annually to monitor progress and advise on the scientific content of the study. In addition, an IDMC will be established to oversee the safety of the trial and a clinical trial monitor will be appointed to ensure that the trial is conducted to GCP standards.

The trial management committee will include the LSHTM PIs, site PIs and the trial administrator. The trial management committee is responsible for overseeing the trial and its members will communicate regularly by teleconferences.

The project will be coordinated by a trial coordinator (to be appointed) who will be based at IRSS/Centre Muraz, Burkina Faso or at the MRTC, USTTB, Mali. He/she will be responsible for running the study under the mentorship of the PIs.

10. Roles of the investigators and collaborators

The team from the IRSS/Centre Muraz, which includes Jean Bosco Ouédraogo, Halidou Tinto and Issaka Zongo will be responsible for conducting the part of the trial undertaken in Burkina Faso and participating in the analysis of the trial results.

The team from MRTC which includes Alassane Dicko, Ogobara Doumbo, and Issaka Sagara will be responsible for conducting the part of the trial undertaken in Mali and participating in the analysis of the trial results.

The LSHTM team (Daniel Chandramohan, Brian Greenwood, Matthew Cairns, Paul Milligan, Simon Cousens, Manuela Claite and Amit Bhasin) will provide epidemiological, statistical, administrative and financial management support.

Lesong Conteh, Imperial College will be responsible for the economic and sociological components of the study.

Statistical support will be given by Matthew Cairns and Paul Milligan who have both worked previously with the teams in Burkina Faso and Mali. However, it is envisaged that the majority of the analyses will be done by the study coordinator and the site investigators.

11. Stakeholders engagement and dissemination plans

Strong links have been established already with the Ministries of Health and NMCPs in Burkina Faso and Mali in connection with the implementation of SMC. These links will facilitate the incorporation of AZ to into SMC regimens if this is found to be a useful intervention. The study teams have established good lines of communication with many organisations during the course of the evaluation of the results of previous SMC trials. These include strong links to WHO Geneva through the MPAC, WHO AFRO, WAHO, UNICEF, The Global Fund, PMI, MSF, the Malaria Consortium and several NMCP managers who attended workshops on SMC held in Philadelphia in 2011 and in Cape Verde in 2012. Thus, if it is found that AZ is a useful addition to SMC regimens, routes have already been established through which this knowledge could be disseminated. Results from the trial will be presented at conferences and

in peer reviewed journals and will be discussed with the study communities at the end of the trial. The data will be shared with the WHO technical expert groups and MPAC.

12. Activity Schedule

Activities	2014								2015								2016								2017	
	J-M	A-J	J	A	S	O	N	D	J-M	A-J	J	A	S	O	N	D	J-M	A-J	J	A	S	O	N	D	J-M	A-J
**1. Project preparation**																										
• Staff recruitment	X	X																								
• Logistics	X	X																								
• Upgrading study health facility	X	X																								
• Ethics & regulatory approvals	X	X																								
• Trial registration		x																								
2. Steering committee meeting	x								x								x								x	
3. IDMC meeting	x								x								x								x	
4. Census and enrolment of children		Xx	x								x															
5. Annual updating of census and enrolment			x								x								x							
6. SMC or SMC+AC rounds				1	2	3	4					1	2	3	4					1	2	3	4			
7. Naso-pharyngeal swabbing[$]				x				x				x				x				x				x		**x**
8. X-sectional malaria & anthropometry survey[@]								x								x								x		
9. Health facility based morbidity & mortality surveillance		X	X	X	X	X	X	X	X	X	X	X	X	X	X	X	X	X	X	X	X	x	X	X		
10. Household level active morbidity & mortality surveillance[£]			x	x	x	x	x	x			x	x	x	x	x	x			x	x	x	x	x	x		
11. Laboratory assays			x	x	x	x	x	x	x	x	x	x	x	x	x	x	x	x	x	x	x	x	x	x		**x**
12. GCP monitoring			x			x							x							x					x	
13. Data management			x	x	x	x	x	x	x	x	x	x	x	x	x	x	x	x	x	x	x	x	x	x	x	
14. Data Analysis & dissemination																									x	**x**

$ In a randomly selected sample of children
@ In a randomly selected sample of children
£ In a randomly selected sample every two week

13. References

[1] Wilson AL. A systematic review and meta-analysis of the efficacy and safety of intermittent preventive treatment in children (IPTc). Plos One 2011; 6: e16976.
[2] Dicko A, Diallo AI, Tembine I et al. Intermittent preventive treatment of malaria provides substantial protection against malaria in children already protected by an insecticide-treated bednet in Mali: a randomised, double-blind, placebo-controlled trial. PLoS Med 2011; 8: 1000407.
[3] Konate AT, Yaro JB, Ouedraogo AZ et al. Intermittent preventive treatment of malaria provides substantial protection against malaria in children already protected by an insecticide-treated bednet in Burkina Faso : a randomised, double-blind, placebo-controlled trial. PLoS Med 2011; 8: e1000408.
[4] Ndiaye JL, Cissé B, Ba EH et al. Safety of seasonal intermittent preventive treatment against malaria with sulfadoxine pyrimethamine + amodiaquine when delivered to children under 10 years of age by district health staff in Senegal. PLoS Med: submitted.
[5] Bojang KA, Akor F, Conteh L, et al. Two strategies for the delivery of IPTc in an area of seasonal malaria transmission in The Gambia: a randomised controlled trial. PLoS Medicine. 2011; 8:e1000409.
[6] Kweku M, Webster J, Adjuik M. et al. Options for the delivery of intermittent preventive treatment for malaria to children: a community randomised trial. PLoS One 2009; 4: e7256.
[7] Greenwood B, Bojang K, Tagbor H, et al. Combining community case management and intermittent preventive treatment for malaria. Trends Parasitol 2011; 27:477-480.
[8] Patoulllard E, Conteh L, Webster J et al. Coverage, adherence and cost of intermittent preventive treatment of malaria in children employing different delivery strategies in Jasikan District, Ghana, PLoS One; 2011; 6 e24871..
[9] Cairns M, Roca-Feltrer A, Garske T, et al. Estimating the public health impact of seasonal malaria chemoprevention in African children. Nat Commun. 2012; 6;3:881.
[10] Milligan, P et al. (2012) Implementing Seasonal Malaria Chemoprevention. Report of a meeting held in Praia, Cape Verde, Sep 7-8 2012.
[11] Berkley JA, Lowe BS, Mwangi I et al. Bacteremia among children admitted to a rural hospital in Kenya. N Engl J Med 2005; 352: 39-47.
[12] Porco TC, Gebre T, Ayele et al. Effect of mass distribution of azithromycin for trachoma control on overall mortality in Ethiopian children. JAMA 2009; 302: 962-968.
[13] Keenan JD, Ayele B, Gebre T et al. Childhood mortality in a cohort treated with mass azithromycin for trachoma. Clin Infect Dis 2011; 52: 883-888.
[14] Cook JA Eliminating blinding trachoma. N Engl J Med 2008; 358:1777-1779.
[15] van Eijk AM, Terlouw DJ. Azithromycin for treating uncomplicated malaria. Cochrane Database of Systematic Reviews 2011, issue 2, CD006688.
[16] (Cutts F, Zaman SM, Enwere G et al. Efficacy of nine-valent pneumococcal conjugate vaccine against pneumonia and invasive pneumococcal disease in The Gambia: randomised, double blind, placebo-controlled trial. Lancet 2005; 356: 1139-46.
[17] Plowe CV, Djimde A, Bouare M, Doumbo O, Wellems TE. Pyrimethamine and proguanil reistance-conferring mutations in plasmodium falciparum dihydrofolate reductase: polymerase chain reaction methods for surveillance in Africa. Am J Trop Med Hyg 1995; 52:565-568.
[18] Ayele B, Gebre T, House JI et al. Short report: adverse events after mass azithromycin treatment for trachoma in Ethiopia. AM J Trop Med Hyg 2011;85:291-294.
[19] Skalet AH, Cevallos V, Ayele B et al. Antibiotic pressure and macrolide resistance in nasopharyngeal *Streptococcus pneumoniae*: a cluster randomized clinical trial. PLoS Med 2010;7: e 10003777.
[20] Batt SL, Charalambous BM, Solomon AW et al. Impact of azithromycin administration for trachoma control on the carriage of antibiotic-resistant Streptococcus pneumoniae. Antimicrob Agents Chemother 2003;47:2765-2769.

APPENDICES

Appendix 1

Calculation of design effect: The average household size in Burkina Faso is 6.6, 18% of whose members will be <5 years of age. Seventy-seven percent of these children, or 0.91 children per household will on average be aged 3-59 months. If the number of children per household is assumed to follow the Poisson distribution, 60% of households will contain at least one child aged 3-59 months, meaning that among households with a child under 5 years, the average is 1.5 children per household. We explored the impact of up to 1.8 children per household on the design effect. Estimates of the intra-cluster correlation (ICC) for mortality from recent DHS data range from 0.17 for Burkina to 0.236 in Mali. By varying these two factors the design effect varies from 1.085 to 1.189. We have, therefore, inflated the sample size assuming an individually randomised trial by 20%.

Number of children aged 3-59 months per household	ICC	Design Effect
1.5	0.17	1.085
1.6	0.17	1.102
1.7	0.17	1.119
1.8	0.17	1.136
1.5	0.236	1.118
1.6	0.236	1.142
1.7	0.236	1.165
1.8	0.236	1.189

Appendix 2

Table 2.1 Sample size estimates shown in the table are inflated by 20% to allow for the design effect (1.2) introduced by household randomization. Estimates that are shown in the sample size section are shaded grey.

Assuming incidence rate of 15 deaths or hospital admissions per 1000				Sample size with 90% power			Sample size with 80% power		
Rate in SMC	Rate in SMC+AZ	efficacy	type I error rate	1 year study	2 year study	3 year study	1 year study	2 year study	3 year study
0.015	0.0105	30%	5%	34,820	18,326	12,849	26,010	13,690	9,598
0.015	0.01065	29%	5%	37,483	19,728	13,831	27,999	14,736	10,331
0.015	0.0108	28%	5%	40,443	21,285	14,924	30,210	15,900	11,148
0.015	0.01095	27%	5%	43,746	23,024	16,143	32,678	17,199	12,059
0.015	0.0111	26%	5%	47,449	24,973	17,509	35,444	18,655	13,079
**0.015**	**0.01125**	**25%**	**5%**	51,615	27,166	**19,046**	38,556	20,293	14,228
0.015	0.0114	24%	5%	56,326	29,645	20,785	42,075	22,145	15,526
0.015	0.01155	23%	5%	61,679	32,463	22,760	46,074	24,249	17,001
**0.015**	**0.0117**	**22%**	**5%**	67,795	35,681	25,016	50,641	26,654	**18,688**
0.015	0.01185	21%	5%	74,823	39,380	27,610	55,891	29,416	20,624
0.015	0.012	20%	5%	82,954	43,660	30,610	61,965	32,613	22,865

Assuming incidence rate of 10 deaths or hospital admissions per 1000				Sample size with 90% power			Sample size with 80% power		
Rate in SMC	Rate in SMC+AZ	efficacy	type I error rate	1 year study	2 year study	3 year study	1 year study	2 year study	3 year study
**0.010**	**0.007**	**30%**	**5%**	52,230	27,490	**19,273**	39,015	20,534	14,396
0.010	0.0071	29%	5%	56,223	29,591	20,746	41,998	22,104	15,498
0.010	0.0072	28%	5%	60,664	31,928	22,385	45,315	23,850	16,721
0.010	0.0073	27%	5%	65,619	34,536	24,214	49,016	25,799	18,088
**0.010**	**0.0074**	**26%**	**5%**	71,173	37,460	26,263	53,165	27,981	**19,618**
0.010	0.0075	25%	5%	77,423	40,749	28,570	57,834	30,439	21,341
0.010	0.0076	24%	5%	84,490	44,469	31,178	63,113	33,218	23,289
0.010	0.0077	23%	5%	92,519	48,694	34,140	69,110	36,374	25,503
0.010	0.0078	22%	5%	101,693	53,523	37,525	75,963	39,980	28,030
0.010	0.0079	21%	5%	112,235	59,071	41,415	83,838	44,125	30,936

Appendix 3: Definitions of adverse and serious adverse events and reporting schedule

3.1. Definition of an adverse event and serious adverse event

An adverse event (AE) is defined as any clinical symptom or sign that occurs in a study child after administration of the study drugs that may or may not have a causal relationship with the study drugs. Examples of an AE include

(1) Occurrence of symptom such as vomiting or diarrhoea in a child who did not have these symptoms prior to the administration of drugs;

(2) unexpected worsening of an existing condition.

A serious adverse event (SAE) is any clinical condition that fulfils at least one of the following criteria:

* results in death,
* results in admission to hospital,
* is life-threatening (the child was at risk of death at the time of the adverse event),
* results in disability/incapacity.

3.2. Severity, relationship of event to study drug, and outcome

The severity of a clinical adverse event is to be scored according to the following scale:

1	Mild:	Awareness of sign or symptom, but easily tolerated.
2	Moderate:	Discomfort enough to cause interference with usual activity.
3	Severe:	Incapacitating with inability to work or perform usual activity.
4	Life-threatening:	Patients at risk of death at the time of the event.
5.	Death	

3.3. Assessment of Causality

The relationship between the study drugs and the occurrence of each AE/SAE will be determined by the project physician in consultation with the site PIs based on their clinical judgment. Alternative causes, such as the natural history of the underlying diseases, concomitant therapy, other risk factors, and the temporal relationship of the event to the study drug will be considered and investigated. The site PIs will consult the lead PIs and the IDMCif this is deemed to be necessary.

There may be situations where the PIs have very minimal information about a SAE to include in the initial report. However, every attempt will be made to make an assessment of causality for every SAE prior to reporting to the IDMC. The PIs may change their opinion of causality in light of follow-up information, and may amend the SAE case report form accordingly.

The relationship of an adverse event to study drug will be assessed according to the following definitions:

1. Definitely unrelated: events that had occurred prior to administration of the study drugs or events that are obviously unrelated to the study (e.g. accidental injury).

2. Unlikely: There is no reasonable temporal association between the study drug and the suspected event and the event could have been produced by the child's clinical state or other concomitant medications.

3. Possible: The suspected adverse event may or may not have a reasonable temporal association with the administration of study drug but the nature of the event is such that an association with the study drug cannot be ruled out. The event could be related to the child's clinical state or by concomitant medications.

4. Probable: The suspected adverse event follows a reasonable temporal sequence after administration of study drugs, abates upon discontinuation of the drug, and cannot be reasonably explained by the known clinical state of the child.

5. Definitely related: events that have no uncertainty in their association to the administration of study drugs.

The outcome of each AE must be assessed according to the following classification:

- Completely recovered : The child has fully recovered with no observable residual effects
- Not yet completely recovered : The child's condition has improved, but still has some residual effects
- Deterioration : The child's overall condition has worsened
- Permanent damage : The AE has resulted in a permanent impairment
- Death : The child died due to the AE
- Ongoing : The AE remains the same as at onset
- Unknown : The outcome of the AE is not known because of lost to follow-up

3.4. Reporting of adverse events and SAEs

All solicited adverse events will be collected in a random sample of children on day 7 post administration of SMC or SMC+AZ using an adverse events record form (AEForm). The AEForm will have a check list of known adverse events, date started, date ended, severity, possible relationship to study drugs, concomitant medications, and action taken.

All serious adverse events will be reported using a SAE report form which will have a detailed narrative of the events including information on the date the event started, severity, possible relationship to study drugs, concomitant medications, action taken, and outcome of the event.

A list of all SAEs will be compiled three-monthly and provided to the IDMC. Any SAE potentially related to drug administration will be reported to the IDMC and institutional Ethics Committees within 48 hours.

The signatures below confirm agreement by the individuals authorised by the sponsor and principal participating institution at the clinical site that the study will be conducted in compliance with protocol version 4 dated 9th July 2014.

....	Ogobara Doumbo	10/07/14
Study Director in Mali	NAME (PRINTED)	DATE
....	....	....
Study Director in Burkina Faso	NAME (PRINTED)	DATE

Les signatures ci-dessous confirment l'accord des personnes autorisées par le sponsor et l'établissement principal participant sur le site clinique où l'étude sera menée en conformité avec le protocole version 4.0 du 03/06/2014

....	....	....
Directeur de l'étude au Mali	Nom	DATE
....	Jean Bosco OUEDRAOGO	17/07/2014
Directeur de l'étude au Burkina Faso	Nom	DATE
